# Editorial: Artificial polyploidy in plants, Volume II

**DOI:** 10.3389/fpls.2022.1124292

**Published:** 2023-01-12

**Authors:** Jeremy E. Coate, Jen-Tsung Chen, Geoffrey Meru

**Affiliations:** ^1^ Department of Biology, Reed College, Portland, OR, United States; ^2^ Department of Life Sciences, National University of Kaohsiung, Kaohsiung, Taiwan; ^3^ Horticultural Sciences Department and Tropical Research & Education Center, University of Florida, Gainesville, FL, United States

**Keywords:** antimitotic agents, artificial polyploidy, plant biotechnology, plant breeding, whole genome duplication

Polyploidy (whole genome multiplication) produces a myriad of dramatic phenotypes, many of which are favored by natural and/or artificial selection. The artificial induction of polyploidy has become an invaluable method both for understanding the evolution of natural polyploid lineages and harnessing polyploidy’s potential to produce traits of value to humans. The 14 studies described in the second volume of the research topic, “Artificial Polyploidy in Plants” highlight new methods for generating polyploids across a wide range of plant lineages, illustrate the range of, and recurring patterns in, polyploid phenotypes, provide new mechanistic insights into polyploid phenotypes, and illustrate the dual utility of inducing genome duplications for understanding plant evolution and improving plant agronomic traits.

## Methods for generating artificial polyploids

To fully harness the utility of synthetic polyploids, it is critical to optimize methods of polyploid induction. Several studies in the current volume tackle this challenge.

Triploids are variously considered to be infertile deadends (triploid block), or important intermediates in the production of tetraploids (triploid bridge). Li et al. demonstrated that triploids in the orchid genus, *Cymbidium*, produce fertile pollen and that crosses involving triploids produce viable offspring of varying ploidies, including stable tetraploids, demonstrating that triploids are more ‘bridge’ than ‘block’ in this genus. This provides support for the idea that triploids can serve as critical intermediates in the evolution of natural polyploids, and illustrates their utility for generating synthetic polyploids.

Commercial garlic is male-sterile and cultivated asexually, limiting the ability to improve traits by traditional breeding methods. Wen et al. established an optimized protocol for *in vitro* induction of autotetraploidy in garlic (*Allium sativum*), achieving induction rates of 21.8% by culturing explants in 0.2% colchicine for 20 days. Additionally, the authors demonstrated that tetraploids exhibited a number of novel traits, including enhanced production of secondary metabolites, and that induction of polyploidy might enhance the crop’s value as a factory for pharmaceuticals.

A major challenge for *in vitro* induction of polyploidy is the frequent formation of unstable mixoploids (cytotypic mosaics). Working with *Magnolia officinalis*, Gao et al. described a novel procedure using colchicine treatment of embryonic cell aggregates to produce, in some cases, 100% tetraploid somatic embryos with no mixoploids. Cell lines and plantlets demonstrated stable ploidy throughout the somatic embryogenesis process, providing a potentially more efficient approach for trait improvement through polyploidy induction.

In addition to the use of antimitotic agents, high-temperature treatments are another method for inducing polyploidy. Liu et al. analyzed the timing of zygotic development in relation to floral bud development in *Eucalyptus urophylla* to identify the optimal timing of heat treatment for inducing zygotic doubling. They first established that zygotic mitosis initiated on day 23 post-fertilization. They then demonstrated, for the first time in *E. urophylla*, that zygotic doubling could be induced by heat treatment on days 24-26, with the highest rate of induction achieved on day 25 (additionally determining the optimal heat treatment conditions for inducing doubling). The authors further documented a range of phenotypic effects in the derived polyploids and linked these traits to changes in gene expression (see below). These data should facilitate the breeding of improved eucalyptus crops *via* polyploidy induction.

## Phenotypic effects of polyploidy

Several studies in this collection illustrate the breadth of phenotypic changes induced by polyploidy. To cite a few examples, polyploids were shown to exhibit novel traits ranging from morphological (e.g., larger cells, thicker leaves, longer wood fibers), physiological (e.g., higher chlorophyll contents and photosynthetic rates) and biochemical (higher contents of secondary metabolites, chlorophyll, and hormones), to complex traits of agricultural significance (higher or lower growth rates, drought tolerance, resistance to lodging).

In some cases, the direction of these phenotypic effects varied by species. For example, though *Cymbopogon khasianus* polyploids were larger than their diploid progenitors (Vimala et al.), polyploids in other taxa exhibited reduced biomass and dwarf phenotypes [e.g., hybrid poplar (Wouters et al.), garlic (Wen et al.), hybrid sweetgum (Chen et al.)]. Often, however, several similar phenotypic effects were observed across evolutionarily distant lineages, revealing some recurring themes in the immediate consequences of polyploidy. Increased cell sizes were documented in garlic (Wen et al.), Cymbopogon (Vimala et al.), and hybrid sweetgum (Chen et al.), and drought tolerance was observed in figs (Abdolinejad and Shekafandeh **)** and sour jujube (Li et al.). Tolerance to drought and other stresses has emerged as a common property of polyploids, including neopolyploids, as discussed by Tossi et al.

A potentially significant source of phenotypic variation in polyploids is alterations in chromosome numbers and gene dosage associated with aneuploidy and structural changes. Zhong et al. used microsatellites and fluorescence *in situ* hybridization to study chromosomal number and structural variation in the offspring of an interploidy cross, *Populus alba* × *Populus berolinensis* ‘Yinzhong’. They identified progeny representing a range of aneuploidies and structural changes which will be a useful resource for future studies on the effects of changes in gene and chromosome dosage on trait variation.

Though numerous studies, including several in this collection, have identified traits that are likely to be beneficial to the plant and/or useful to humans, it is important to keep in mind that the effects of polyploidy are, in many cases, highly variable, taxon-specific, and/or deleterious (or at least undesirable from a human-use point of view). Wouters et al. demonstrated that induction of polyploidy in hybrid poplar resulted in a propensity for their apical meristems to break, as well as in reduced biomass and increased lignin content (resulting in lower saccharification efficiency). Though this contrasted with what was observed previously in Arabidopsis, the reduced biomass was consistent with what Wen et al. observed in Allium, and what Liu et al. reported for *Eucalyptus urophylla*. Thus, just as polyploidy has mixed effects on evolutionary success in natural populations, these studies serve as a reminder that polyploidy should not be oversold as a panacea for crop improvement.

## Molecular phenotypes and mechanistic insights into the consequences of polyploidy

Though numerous studies have documented a plethora of phenotypes associated with polyploidy, our understanding of the underlying molecular mechanisms lags behind. Several studies in the current collection contribute important insights on this front as well. Tossi et al., for example, undertook a comprehensive review of the mechanisms by which polyploidy confers stress tolerance. Among many mechanisms discussed, they note that epigenetic modifications and changes in hormone levels have emerged as key players in polypoid phenotypes (including tolerance to various stresses), and examples of both are reported in this collection.

Xiao et al. characterized changes to the DNA methylome of autotetraploid cassava (*Manihot esculenta*) and concluded that variation in methylation around transposable elements acts to adaptively modulate dosage responses of protein-coding genes and lncRNA genes. Abdolinejad and Shekafandeh showed that enhanced water stress tolerance in figs was associated with elevated levels of stress hormones. Chen et al. showed that the dwarf phenotype in tetraploid hybrid sweetgum (*Liquidambar styraciflua × L. formosana)* correlated with differential expression of hormone biosynthetic genes, and with reduced levels of auxin, gibberellin, and brassinolide in both roots and stems. Notably, they showed that exogenous gibberellin and auxin treatments significantly promoted stem and root growth, and concluded that reduced growth in the tetraploids was a consequence of auxin and gibberellin deficiency. Interestingly, Tossie et al. describe cases in which stress response genes, including those involved in jasmonic acid biosynthesis, are primed by hypomethylation, enabling a more rapid and robust response to stress. Thus, epigenetic modifications and hormone biosynthesis have been mechanistically linked in promoting stress tolerance in polyploids.

The molecular changes that contribute to polyploid phenotypes extend beyond epigenetics and hormone biology, however. Abdolinejad and Shekafandeh, for example, showed that in addition to elevated hormone levels, drought tolerance in figs was associated with elevated levels of protective osmolytes and scavengers of reactive oxygen species, suggesting that tetraploidy induces a multi-layered stress response.

Two studies in this collection link polyploid phenotypes to transcription factors. Li et al. showed that the enhanced drought tolerance in sour jujube autotetraploids correlated with a near doubling in vessel elements and parenchyma cells and that the transcription factor, ZjVND7, which is known to play a central role in xylem vessel development, was differentially expressed between diploids and tetraploids under drought conditions. Using DAP-Seq, they found more targets of ZjVND7 in the tetraploid, with enrichment for targets involved in xylem development. Among these targets was the gene *ZjSMR1*, which had a stronger binding signal for ZjVND7 in tetraploids than in diploids under drought. They hypothesize that altered ZjVND7 activity upregulates ZjSMR1 to promote xylem vessel formation. Liu et al. found that differences in leaf chlorophyll contents between diploid and triploid *E. urophylla* correlated with changes in the expression of Golden 2-Like (GLK) transcription factors, and provided evidence that GLKs positively regulate gene co-expression networks involved in chlorophyll biosynthesis.

## Conclusions and perspectives

Polyploidy induces a range of traits of value to humans, and inducing polyploidy has become an important tool in the plant breeder’s toolbox (Chen et al., 2020). At the same time, generating synthetic polyploids allows researchers to step back in time to better understand the early evolution of natural polyploids. As with volume I of this Research Topic, volume II highlights several recent advances in the biology of artificial polyploidy. The 14 studies included in this volume highlight the very active and vibrant nature of ongoing research in this field, describing advances in our ability to generate polyploids, as well as in our understanding of polyploid phenotypes and how they arise. We hope this collection stimulates further work and breakthroughs on this important topic.

## Author contributions

J-TC, JC and GM drafted the manuscript. All authors revised and approved the final version.
